# Enhancing the Conformational Stability of the cl-Par-4 Tumor Suppressor via Site-Directed Mutagenesis

**DOI:** 10.3390/biom13040667

**Published:** 2023-04-12

**Authors:** Samjhana Pandey, Krishna K. Raut, Antoine Baudin, Lamya Djemri, David S. Libich, Komala Ponniah, Steven M. Pascal

**Affiliations:** 1 Biomedical Sciences Program, Old Dominion University, Norfolk, VA 23529, USA; spand003@odu.edu; 2 Department of Chemistry and Biochemistry, Old Dominion University, Norfolk, VA 23529, USA; kraut001@odu.edu (K.K.R.); ldjem001@odu.edu (L.D.); kponniah@odu.edu (K.P.); 3 Greehey Children’s Cancer Research Institute, University of Texas Health Science Center at San Antonio, San Antonio, TX 78229, USA; baudin@uthscsa.edu (A.B.); libich@uthscsa.edu (D.S.L.); 4 Department of Biochemistry and Structural Biology, University of Texas Health Science Center at San Antonio, San Antonio, TX 78229, USA

**Keywords:** intrinsically disordered proteins (IDPs), prostate apoptosis response-4 (Par-4), tumor suppressor, site-directed mutagenesis, circular dichroism (CD) spectroscopy, dynamic light scattering (DLS), nuclear magnetic resonance (NMR) spectroscopy

## Abstract

Intrinsically disordered proteins play important roles in cell signaling, and dysregulation of these proteins is associated with several diseases. Prostate apoptosis response-4 (Par-4), an approximately 40 kilodalton proapoptotic tumor suppressor, is a predominantly intrinsically disordered protein whose downregulation has been observed in various cancers. The caspase-cleaved fragment of Par-4 (cl-Par-4) is active and plays a role in tumor suppression by inhibiting cell survival pathways. Here, we employed site-directed mutagenesis to create a cl-Par-4 point mutant (D313K). The expressed and purified D313K protein was characterized using biophysical techniques, and the results were compared to that of the wild-type (WT). We have previously demonstrated that WT cl-Par-4 attains a stable, compact, and helical conformation in the presence of a high level of salt at physiological pH. Here, we show that the D313K protein attains a similar conformation as the WT in the presence of salt, but at an approximately two times lower salt concentration. This establishes that the substitution of a basic residue for an acidic residue at position 313 alleviates inter-helical charge repulsion between dimer partners and helps to stabilize the structural conformation.

## 1. Introduction

Intrinsically disordered proteins/protein regions (IDPs/IDRs) do not follow the conventional sequence-structure-function paradigm [1,2]. These proteins lack a well-defined tertiary structure under physiological conditions, but many can form structures or structural ensembles under certain conditions [2]. The propensity of these proteins to switch conformation upon either interaction with ligands, post-translational modification, or change in ionic strength or pH, allows them to play crucial roles in cell signaling and regulation [3,4,5,6,7,8,9]. Many IDPs/IDRs have been reported to be associated with human diseases such as cardiovascular disease, neurodegenerative disease, diabetes, and cancer [10,11,12,13].

**Figure 1:**
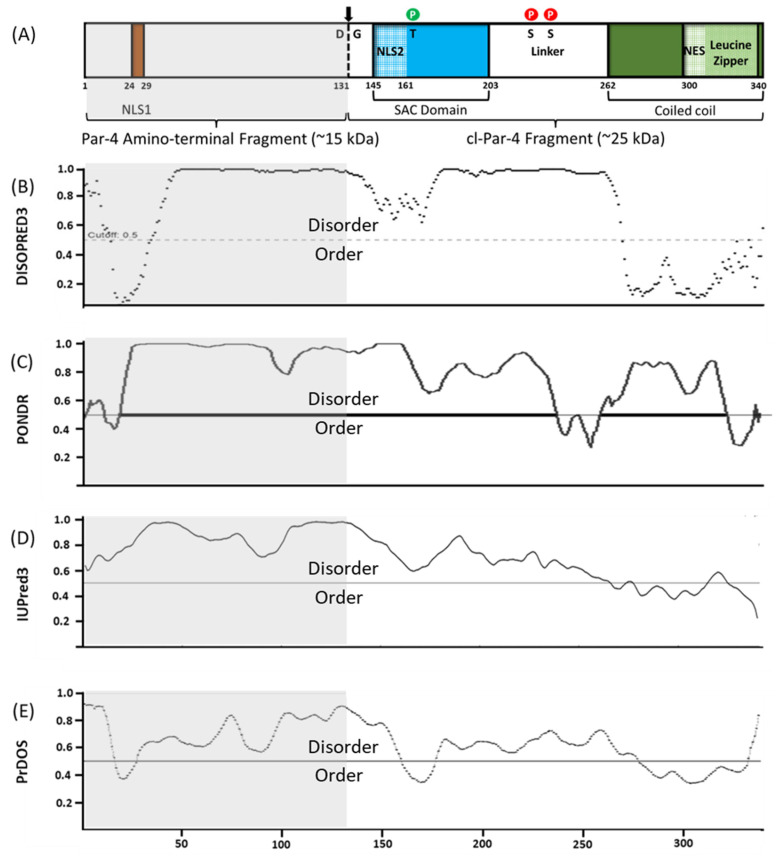
(**A**) Domain diagram of full-length Par-4 and (**B**–**E**) disorder prediction of the full-length Par-4 sequence using: (**B**) DISOPRED3 [15,16], (**C**) PONDR [17], (**D**) IUPred3 [18], and (**E**) PrDOS [19].

Prostate apoptosis response-4 (Par-4), a proapoptotic tumor suppressor protein, is a predominantly intrinsically disordered protein [14]. This experimental finding is consistent with its amino acid composition: in Par-4, polar and charged residues such as aspartic acid, glutamic acid, lysine, and arginine residues are found in abundance, while bulky hydrophobic residues are less abundant [14]. Order/disorder analysis of full-length Par-4 based on amino acid sequence therefore predicts between approximately 65% and 90% disorder (Figure 1) depending on the algorithm used.

Par-4 expression in healthy cells is ubiquitous while its expression is down-regulated in a variety of cancers such as prostate, breast, renal, and neuroblastoma [20,21,22,23,24]. In contrast, over-expression of this protein has been found to be positively associated with the development of several neurodegenerative disorders [25,26,27]. Par-4 selectively induces apoptosis in cancer cells [28]. This selectivity is primarily due to two targeting factors. First, extrinsic Par-4 enters cells by binding to the surface protein receptor GRP78; GRP78 levels are elevated in cancer cells [29]. Second, Par-4 must be phosphorylated by PKA at T163 in order to enter the nucleus; PKA activity is also elevated in cancer cells [22,30].

When apoptosis is activated, full-length Par-4 is cleaved after Asp-131 by caspase-3 to generate two fragments: (i) the 15 kilodalton PAF (Par-4 amino-terminal fragment) and (ii) the 25 kilodalton cl-Par-4 (cleaved Par-4) as shown in Figure 1A [31]. The active fragment, cl-Par-4, translocates to the nucleus where it inhibits NF-κB and Bcl-2-mediated cell survival pathways required by many cancer cells [32,33,34]. The PAF fragment is believed to be involved in the rescue of full-length Par-4 from Fbxo45-mediated degradation [35,36]. 

Like full-length Par-4, the cl-Par-4 fragment contains a high percentage of polar and charged amino acids and a low content of hydrophobic residues. However, the analysis shown in Figure 1 indicates that the cl-Par-4 fragment has higher order propensity than the PAF fragment. Most notably, the C-terminal 80 residues of cl-Par-4 are known to form a coiled coil (CC), at least under certain conditions [14,37,38,39]. Even in the CC, the order propensity is not strong. This is consistent with the fact that CC regions in their monomeric state are typically disordered and only fold upon dimerization [40]. Thus, a coiled coil can be thought of as an equilibrating order/disorder region, and conditions that promote dimerization concomitantly promote order. Other regions of cl-Par-4 appear to have some order propensity, or at least weak disorder propensity. These include portions of the SAC domain [28], which is the minimal region needed to trigger apoptosis in certain cells, and portions of the linker that connects the SAC domain to the CC.

Our previous studies have shown that cl-Par-4 achieves a compact, highly helical (approximately 80% helix), and stable conformation in the presence of either high salt or acidic pH [37,38]. We have also demonstrated that cl-Par-4 shows a similar conformation in the presence of divalent cations as it does in the presence of monovalent cations, but at a five times lower cation concentration [39]. This requirement of high salt or low pH could be due to the existence of inter-helical electrostatic repulsion between charged residues at the leucine zipper dimer interface near the C-terminus [41,42].

To test this hypothesis, we constructed, expressed, and purified a D313K cl-Par-4 mutant to attempt to reduce the above-mentioned inter-helical charge repulsion and perhaps replace it with an inter-helical salt bridge that stabilizes the conformation. We investigated the cl-Par-4 D313K conformation vs. salt concentration and vs. pH and compared the results to those of the WT cl-Par-4. We employed circular dichroism (CD) spectroscopy, dynamic light scattering (DLS), and nuclear magnetic resonance (NMR). The data show that D313K behaves in many ways like the WT: it forms soluble aggregates in the presence of low salt at neutral pH and acquires a compact, folded, and stable conformation in the presence of high salt or low pH. However, the D313K mutant converts from soluble aggregates to soluble, highly helical, and compact tetramers at a lower salt concentration than is required by the WT. This confirms that the D313K mutation, by reducing inter-helical charge–charge repulsion, stabilizes the compact helical conformation of cl-Par-4. 

## 2. Materials and Methods

### 2.1. Expression and Purification of the D313K cl-Par-4 Mutant

The cl-Par-4 mutant (D313K) construct was prepared by site-directed mutagenesis [43] using a modified expression vector, H-MBP-3C [44], containing codon-optimized (for *E. coli* expression) human wild-type cl-Par-4 (132–340) as a template. In the process, the template DNA was PCR-amplified in two separate tubes using separate mutant forward (**5′-GACCTGCTGAACCGTAAACTGGACGATATTGAAG-3′**) and mutant reverse (**5′-CTTCAATATCGTCCAGTTTACGGTTCAGCAGGTC-3′**) primers (Eurofins Genomics, Louisville, KY, USA). Then, reaction mixtures from the forward and reverse primer tubes were combined and denatured at 95 °C for 5 min, then gradually cooled to 37 °C. The (methylated) non-mutated parental strands were removed by overnight digestion with *DpnI* enzyme. The mutant construct was purified using a Wizard SV Gel and PCR Clean-Up System kit (Promega, Madison, WI, USA) and verified via DNA sequencing. Sequence-verified plasmids containing the D313K mutant were then transformed into electrocompetent BL21 (DE3) codon plus *E. coli* cells. 

Expression and purification of D313K were conducted following the established procedure for WT cl-Par-4 [37,39]. In brief, BL21 (DE3) codon plus *E. coli* cells, transformed with the codon-optimized mutant construct D313K, were cultured in Luria-Bertani (LB) medium supplemented with ampicillin for selective growth at 37 °C. The cells were induced for protein expression by adding 0.5 mM of isopropyl thio-β-D-galactoside (IPTG) when an optical density (OD_600_) of 0.8–0.9 was reached. The induction was followed by further growth at a reduced temperature of 15 °C until an OD_600_ of 1.5–1.6 was reached. The cells were then harvested and lysed and the expressed protein was purified via immobilized metal affinity chromatography using an HisTrap HP 5 mL Ni column (GE Healthcare, Uppsala, Sweden) attached to an AKTA pure chromatography system (GE Healthcare, Uppsala, Sweden). The H-MBP tag was removed by cleavage using a His-tagged 3C-protease enzyme [44]. The purified D313K protein was then dialyzed against a high-salt storage buffer (10 mM Tris, 1 M NaCl, 1 mM TCEP, pH 7.0). In order to improve the precision of the sample concentrations, the protein was concentrated to approximately 10 mg/mL (using a 10 kDa MWCO Vivaspin Turbo 15 centrifugal concentrator; Sartorius, Epsom, UK) and then diluted to 0.2 mg/mL for data analysis. Protein samples that were not immediately used were lyophilized for storage and later reconstituted with sterile DI water when needed. Tests show identical behavior before and after lyophilization.

### 2.2. Expression and Purification of Isotopically (^15^N) Labeled WT & D313K

Expression and purification of isotopically labeled WT and D313K were conducted using a similar procedure as the one used for the unlabeled D313K, with slight modification. BL21 (DE3) codon plus *E. coli* cells transformed with a codon-optimized construct of WT or D313K were first grown in LB medium supplemented with ampicillin for biomass production until an OD_600_ of 0.6–0.8 was reached. The cells were then harvested, washed with M9 salt solution (without carbon and nitrogen sources), resuspended in isotopically (^15^N) labeled M9 minimal medium, and incubated at 37 °C for an hour in a shaking incubator to allow the recovery of growth and the clearance of unlabeled metabolites. After an hour of incubation, the cells were induced for protein expression by adding IPTG to a concentration of 0.5 mM, followed by overnight (approximately 16 h) incubation at 15 °C until the OD_600_ reached 1.4–1.6. Purification of isotopically labeled WT and D313K was conducted following the same method as used for the purification of unlabeled D313K, except the proteins were never lyophilized. The purified and concentrated proteins were stored at 4 °C in a high-salt storage buffer (10 mM Tris, 1 M NaCl, 1 mM TCEP) of pH 7.0.

### 2.3. Circular Dichroism Spectroscopy

The secondary structure of the protein was analyzed by recording CD spectra on a J-815 CD spectrometer (Jasco, Easton, MD, USA) in the far-UV region (195–260 nm wavelength). For all CD samples, the protein concentration was adjusted to 0.2 mg/mL (0.0083 mmol/L). Each sample contained approximately 20–40 mM NaCl as a residual salt from dilution of the high-salt storage buffer (10 mM Tris, 1 M NaCl, 1 mM TCEP, pH 7.0), which is excluded from the calculation of final salt concentrations for simplicity. A single scan was recorded at a scan speed of 20 nm/min using a 1 mm pathlength quartz cuvette at 25 °C. Respective buffer blanks were used for each of the samples and were later subtracted from the sample spectra. The resultant spectra were smoothed using a means-movement function of 25 nm. To easily compare the spectra, the intensities of all the CD spectra were reported in molar ellipticity units. 

Similarly, to study the effect of pH on D313K cl-Par-4 and the WT, samples were prepared at a concentration of 0.2 mg/mL using Tris buffer of varying pH (4.0, 5.0, and 7.0). However, the WT samples were in 10 mM MgSO_4_ whereas the D313K samples were in 10 mM NaCl buffers.

### 2.4. Dynamic Light Scattering

Hydrodynamic radii (Stokes radii) of the protein particles in solution were measured by dynamic light scattering (DLS) using a NanoBrook Omni particle sizer and zeta potential analyzer (Brookhaven Instruments Corporation, Holtsville, NY, USA). Sample preparation followed a similar procedure to the one used for CD spectroscopy: the protein concentration was adjusted to 0.2 mg/mL. DLS data were recorded at ambient temperature using a disposable plastic cuvette of 1 cm pathlength, a standard laser diode laser at 640 nm wavelength, and a scattering angle of 173°. A total of five scans were recorded for each of the samples and hydrodynamic radii for the size of the protein particles were calculated from the mean effective diameter obtained from the summary statistical report of the NanoBrook software. Some outlier values in hydrodynamic diameter from the five-scan series were excluded from the dataset, and mean diameters and radii were recalculated manually from the remaining data points.

Sample preparation for the study of the effect of pH was conducted as described above for the CD samples.

### 2.5. Isotopically (^15^N) Labeled WT/D313K Sample Preparation for NMR

We previously found that cl-Par-4 at pH 7.0 requires a high salt concentration to form compact helical tetramers [38,39]. For NMR analysis, high salt and tetramer formation are problematic in terms of the signal-to-noise ratio and line width. Therefore, for NMR analysis, we employed the conditions of pH 4, which we have shown can induce a compact helical dimer at low salt concentration [37]. To change sample conditions to pH 4.0 with low salt while avoiding precipitation near the protein pI of 5.39, a step-wise dilution/titration procedure was used: 1.0 mL of concentrated (>10 mg/mL) cl-Par-4 in pH 7.0 buffer (10 mM Tris, 1 M NaCl, 1 mM TCEP, pH 7.0) was added dropwise to a solution of 49.0 mL of pH 3.9 buffer (10 mM Tris, 10 mM NaCl, 1 mM TCEP, pH 3.9) with constant stirring. The result was a 50 mL solution of diluted cl-Par-4 (~0.2 mg/mL) at pH 4.0 with 30 mM NaCl. This was then filtered using a 0.22 µm steriflip (Millipore Steriflip, Millipore-Sigma, Darmstadt, Germany), followed by concentration of the protein to 0.5 mM (12 mg/mL) using a 10 kDa MWCO Vivaspin Turbo 15 centrifugal protein concentrator (Sartorius, Epsom, UK). To prevent precipitation upon the addition of D_2_O to the pH 4.0 cl-Par-4 NMR samples, D_2_O was titrated to pH 4 in NMR buffer before being added to the NMR samples for deuterium lock purposes.

### 2.6. NMR Spectroscopy

NMR spectra were recorded on a Bruker Avance NEO spectrometer operating at a proton Larmor frequency of 700.13 MHz using a 5 mm TCI z-axis gradient cryogenic probe. All spectra were recorded at a temperature of 298 K. Uniformly ^15^N-labeled WT or D313K samples were used at a concentration of 0.5 mM, with 7% D_2_O added for locking purposes. ^1^H,^15^N-HSQC spectra were recorded with 64* and 1024* complex points in indirect (^15^N) and direct (^1^H) dimensions, respectively, with acquisition times of 41 ms and 112.6 ms, respectively, for the WT protein. Similar parameters were used for the D313K mutant but with an acquisition time of 37.5 ms in the indirect dimension (^15^N). For both spectra, the data were apodized with a sine bell function and zero-filled to twice the size of the recorded matrix for data analysis. All spectra were initially processed with TopSpin 4 software and further analyzed with CcpNmr analysis 3.1.0 [45].

## 3. Results

### 3.1. Effect of the D313K Mutation on CD Spectra

**Figure 2:**
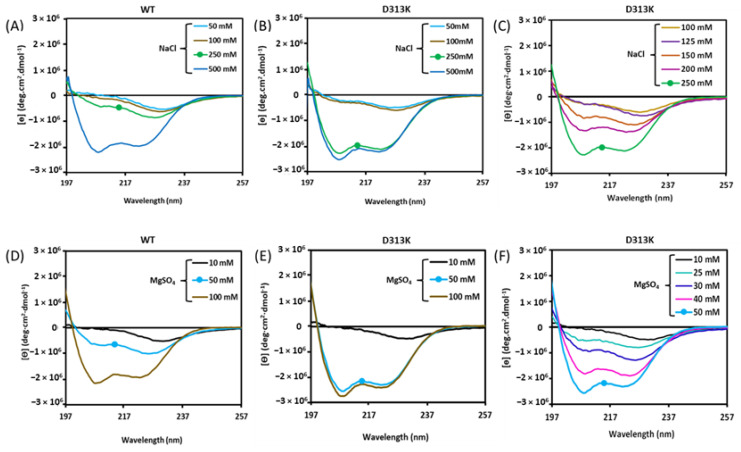
CD spectra of cl-Par-4 as a function of cation concentration for (**A**) WT with Na^+^ [39]; (**B**,**C**) D313K with Na^+^; (**D**) WT with Mg^2+^ [39]; and (**E**,**F**) D313K with Mg^2+^. The intermediate salt concentrations (250 mM NaCl and 50 mM MgSO_4_) for which the WT and mutant CD spectra differ the most are marked with a dot. All data were acquired at pH 7.0 and 25 °C.

Previously, we reported concentration-dependent effects of salts on the CD spectra of WT cl-Par-4. An intense spectrum characteristic of ~80% alpha-helix content (with two intensity minima at 208 and 222 nm wavelengths) was found in the presence of 500 mM or higher concentrations of either of the monovalent cations sodium or potassium (Figure 2A) [38,39]. The spectral intensity decreases with decreasing salt concentration, with an intermediate intensity at 250 mM salt. At high salt concentration, the θ_222_/θ_208_ ratio is less than one, which is consistent with non-coiled coil helices [46]. Here, D313K cl-Par-4 shows a similar trend as the WT: decreasing CD intensity and increasing θ_222_/θ_208_ ratio with decreasing NaCl (Figure 2B). However, the results are shifted to lower salt concentrations. For instance, at 250 mM NaCl, the WT spectrum is not intense, while the D313K spectrum is almost completely converted into the highly helical spectrum characteristic of the well-folded tetramer. The behavior of D313K at intermediate salt concentrations (Figure 2C) shows this trend clearly. These results indicate that the D313K protein requires approximately half the amount of salt as the WT to produce a high-intensity helical CD spectrum.

We also investigated the effect of a divalent cation (Mg^2+^) on the structure of D313K and compared the results to that of the WT. We previously showed that magnesium has a similar effect as sodium on WT cl-Par-4 but at an approximately 5-fold lower ion concentration (Figure 2D) [39]. Here, we found that D313K responded similarly to magnesium compared to the WT but at an additional 2-fold lower ion concentration (Figure 2E). For instance, 50 mM MgSO_4_ produces an intermediate spectrum for the WT but produces a high intensity spectrum resembling the helical tetramer conformation for D313K. The behavior of D313K at intermediate magnesium concentrations (Figure 2F) shows this trend clearly. Again, the θ_222_/θ_208_ ratio for the high intensity spectra is less than one, indicating non-coiled coil helix formation. These results are in harmony with the results obtained with the monovalent cation and indicate that D313K requires approximately half the amount of salt as the WT to produce the compact helical conformation, regardless of whether the salt contains monovalent or divalent cations.

### 3.2. Effect of the D313K Mutation on Particle Size

**Figure 3:**
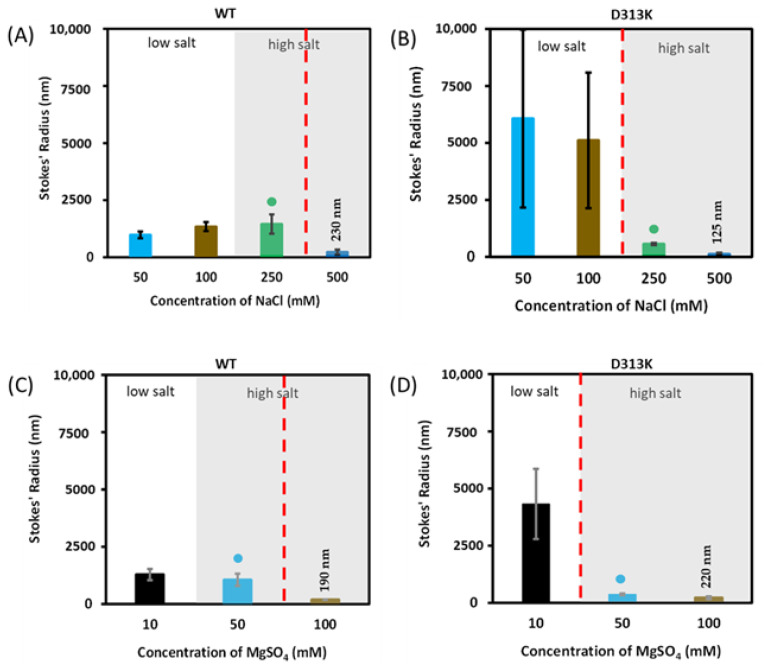
Hydrodynamic size of cl-Par-4 as a function of cation concentration for (**A**) WT with Na^+^ [39]; (**B**) D313K with Na^+^; (**C**) WT with Mg^2+^ [39]; and (**D**) D313K with Mg^2+^. All data were acquired at pH 7.0 and ambient temperature. High salt conditions are indicated by a shaded background. The demarcation line between large and small particles is indicated by a dashed red line. The intermediate salt concentrations (250 mM NaCl and 50 mM MgSO_4_) for which the WT and mutant hydrodynamic radii differ the most are marked with a dot. The smallest hydrodynamic radii are at the highest salt concentration in each panel (**A**–**D**): 230 ± 10 nm, 125 ± 60 nm, 190 ± 5 nm, and 220 ± 60 nm, respectively.

The size of the particles in solution provides an indirect means of measuring conformational changes in proteins, at least in terms of the self-association state. Here, we compared the hydrodynamic sizes of WT cl-Par-4 (Figure 3A) with those of D313K (Figure 3B) under varying salt conditions using dynamic light scattering (DLS). We found that the D313K particles were substantially larger than the WT particles under low salt conditions, but significantly smaller under high salt conditions (Figure 3A,B). In each panel, a red dashed demarcation line is drawn to indicate the transition from large to small particles. Note that the demarcation line shifts to lower salt concentrations for the D313K data. These data are consistent with the CD spectra of Figure 2, in that high CD intensity correlates with small particle size. A similar trend is seen with the divalent cation magnesium (Figure 3C,D): D313K produces larger particles than WT at low MgSO_4_, but smaller particles at high MgSO_4_. Again, the demarcation line shifts to lower salt concentration for D313K, and small particle size (Figure 3D) corresponds to high-intensity CD spectra (Figure 2E). 

### 3.3. Effect of the D313K Mutation on CD Spectra and Particle Size at Low Salt as a Function of pH

**Figure 4:**
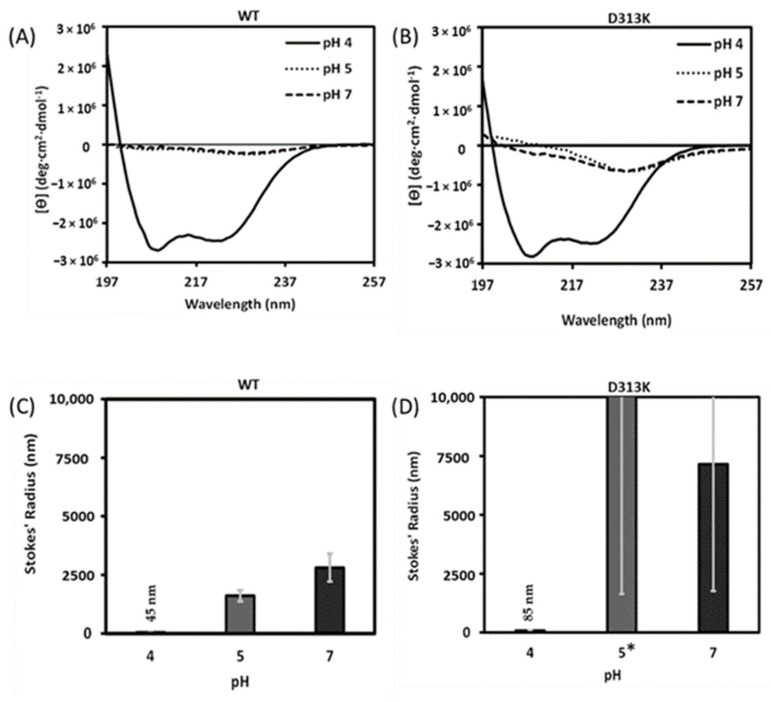
Effect of pH on low salt samples. (**A**) CD spectra of WT; (**B**) CD spectra of D313K; (**C**) hydrodynamic radii of WT (the Stokes radius at pH 4 is 45 ± 1 nm); and (**D**) hydrodynamic radii of D313K (the Stokes radius at pH 4 is 85 ± 15 nm). The asterisk indicates that the hydrodynamic radius at pH 5 extends off the graph to 17,800 nm. The WT samples contain 10 mM MgSO_4_ while the D313K samples contain 10 mM NaCl. All data were recorded at ambient temperature.

WT cl-Par-4 produces CD spectra characteristic of high helicity at pH 4, even in the presence of only low salt concentration (Figure 4A) [37]. Low salt with higher pH results in a drastic reduction in CD intensity (Figure 4A) and large particle size (Figure 4C). A similar trend was seen with D313K, with intense CD spectra and small particle size at pH 4 and low salt (Figure 4B,D, respectively) and even larger particle size than the WT at higher pH (Figure 4D). These results show that the D313K mutant retains the pH dependence that was found with the WT cl-par-4, particularly in terms of particle size reduction and secondary structure at pH 4.

### 3.4. Effect of the D313K Mutation on NMR Spectra

**Figure 5:**
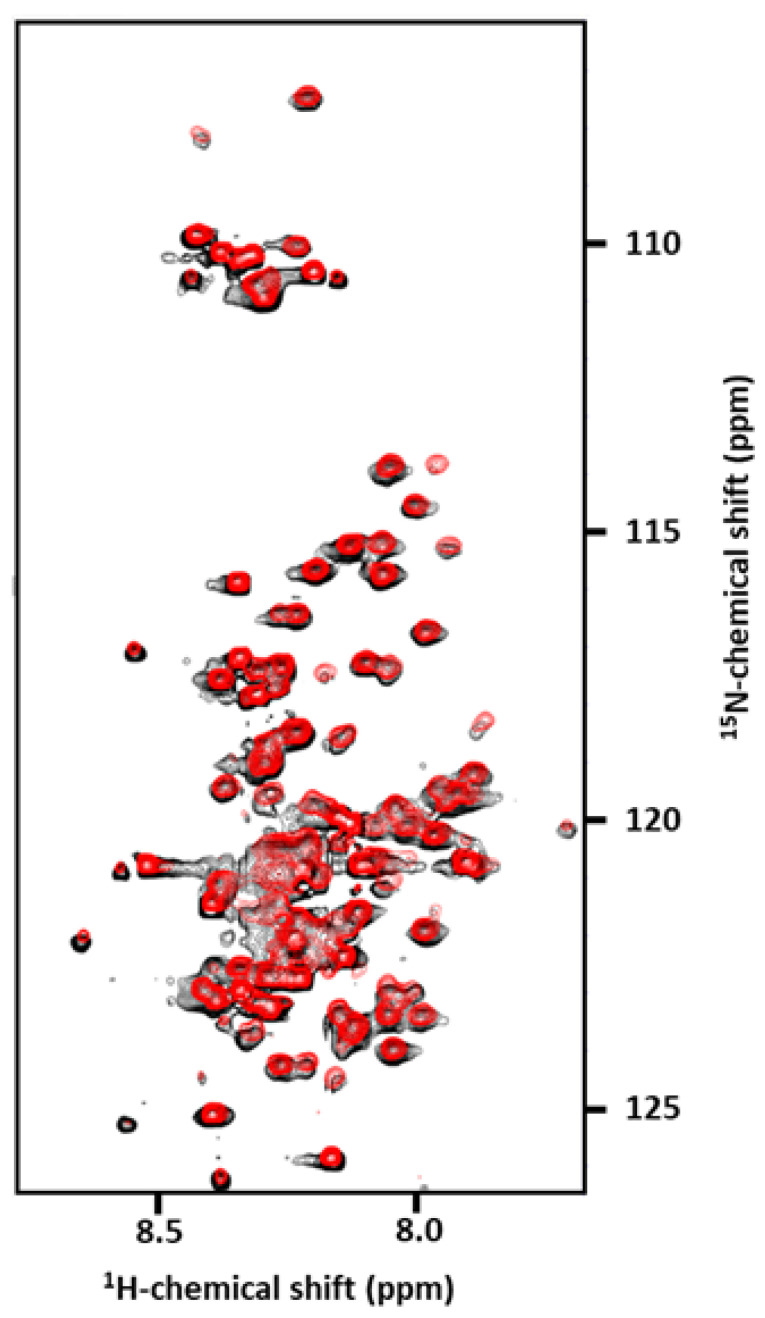
Overlay of ^1^H,^15^N-HSQC spectra of WT (black contours) and D313K (red contours). Spectra were recorded on a 700 MHz spectrometer with a cryoprobe. Sample conditions were 0.5 mM protein with 10 mM Tris, 30 mM NaCl, and 1 mM TCEP, at pH 4.0 and a temperature of 298 K.

To monitor the effect of the D313K mutation on tertiary structure, ^1^H,^15^N-HSQC spectra were obtained with uniformly ^15^N-labeled WT and D313K cl-Par-4 at pH 4 (Figure 5). The nearly perfect overlay of the two spectra provides no indication of change in tertiary structure. This is consistent with the CD and DLS results of the previous section that indicate that the WT and D313K proteins have similar secondary structures and particle sizes at pH 4.

## 4. Discussion

### Influence of Salts on D313K vs. WT

**Figure 6:**
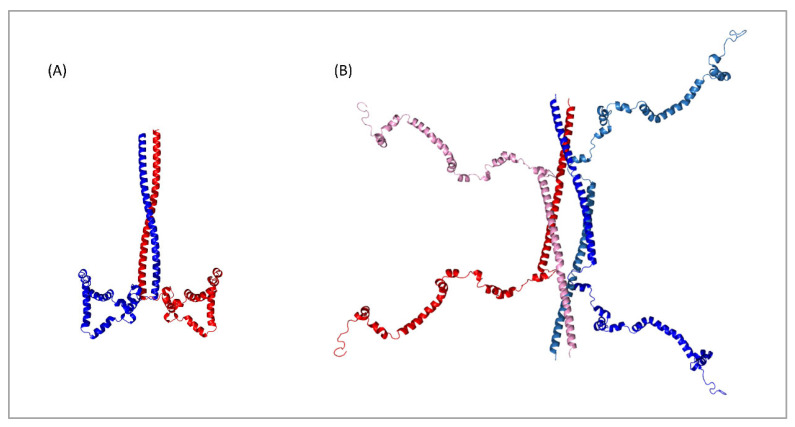
Structural models of cl-Par-4. (**A**) Dimer form, consistent with acidic pH data. Model generated using GalaxyTBM on the GalaxyWEB server using rat Par-4 CC domain crystal structure (pdb 5fiy_A) as a template and allowing the remainder of the protein to fold computationally [37,48,49]; (**B**) Tetramer form, consistent with high salt data. Model generated using GALAXYWEB HOMOMER on the GalaxyWEB server using pdb 5DOL (YabA) as a template for the four central helices and allowing the remainder of the protein to fold computationally [38,48,50]. Each color represents a distinct monomer.

**Figure 7:**
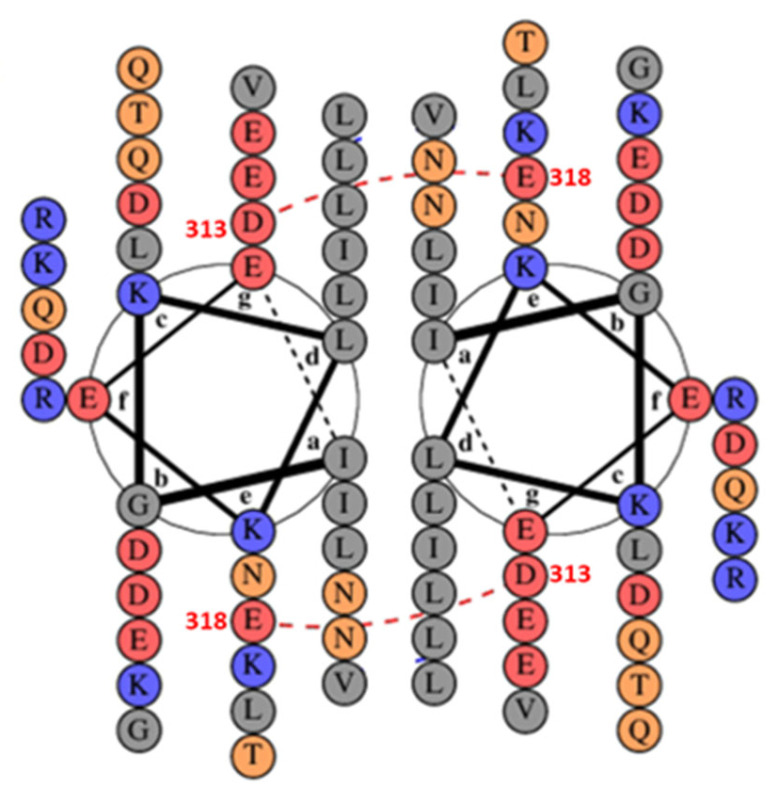
Helical wheel diagram of human Par-4 LZ dimer. The red dashed line indicates inter-helical charge repulsion between the two acidic residues D313 and E318.

The approximately 25 kDa C-terminal fragment of the Par-4 tumor suppressor that is released by caspase-induced cleavage (cl-Par-4) is an active fragment that plays a key role in apoptosis induction in cancer cells [31,47]. Sequence analysis suggests disorder in 60–85% of cl-Par-4 depending upon the prediction tool used (see Figure 1), with the highest order propensity in the coiled coil region, which comprises less than 40% of cl-Par-4. However, CD spectra and scattering data under in vitro conditions of acidic pH or high salt show an approximately 80% helical and compact conformation of dimers or tetramers, respectively [37,38,39]. Structural models of the dimer and tetramer conformations are provided in Figure 6. This high helical content clearly indicates that, at least under these conditions, alpha helix forms in cl-Par-4 outside of the CC region. The most likely site of this additional helicity is in the SAC domain, which shows some order propensity based on sequence analysis (see Figure 1). At pH 7, a monovalent cation (NaCl or KCl) concentration of 500 mM or a divalent cation (MgCl_2_ or MgSO_4_) concentration of 100 mM is required to produce the helical tetramer [38,39]. Based on previous mutagenic analysis of the isolated leucine zipper (LZ) of rat Par-4 [41], we hypothesized that charge–charge repulsion between D313 and E318 in the LZ of human Par-4 (see Figure 7) is at least partly responsible for the salt and pH sensitivity of cl-Par-4.

To test this hypothesis, we created a D313K mutant of human cl-Par-4, which eliminates the possibility of D313–E318 charge repulsion (see Figure 7) and potentially replaces it with a salt bridge. The D313K cl-Par-4 mutant was tested for pH and salt sensitivity. Analyses of the CD spectra and DLS data show that D313K cl-Par-4 forms approximately 80% helical particles under high salt and low pH conditions, similar to the behavior of WT cl-Par-4 (see Figure 2). However, the threshold for high salt is shifted to an approximately two-fold lower concentration. That is, 250 mM NaCl or 50 mM MgSO_4_ are sufficient to induce the intense helical CD spectra that are seen only at twice this salt concentration with WT cl-Par-4.

DLS data of D313K at these conditions (see Figure 3) indicate particle sizes (~565 nm at 250 mM NaCl and ~375 nm at 50 mM MgSO_4_) that are approximately three-fold smaller than the WT cl-Par-4 particles (~1450 nm at 250 mM NaCl and ~1050 nm at 50 mM MgSO_4_). However, D313K hydrodynamic radii consistent with the tetramer (~200 nm) were obtained only at higher salt concentration (500 mM NaCl or 100 mM MgSO_4_). Smaller particle sizes, consistent with dimer formation, were obtained at pH 4 for the WT (~45 nm) and D313K (~85 nm), although a slightly higher value was seen for D313K (see Figure 4).

CD spectroscopy is a sensitive probe of secondary structure, but the potential exists that tertiary interactions could be disrupted by mutation [51,52,53]. Therefore, NMR spectroscopy was used to further analyze the effect of the D313K mutation. Nearly identical ^1^H,^15^N-HSQC spectra show that under the NMR spectroscopy conditions, little, if any, difference is detected between the WT and D313K cl-Par-4 three-dimensional structures (see Figure 5). Due to superior NMR spectral characteristics at pH 4 and low salt relative to pH 7 and high salt, the NMR studies were performed at pH 4. The high salt required for the formation of small particles at pH 7 reduces the intensity of NMR spectra, particularly for cryoprobes, due to effects on tuning, matching, pulse length, and conductivity. Moreover, tetramer formation (at pH 7) produces an approximately 100 kilodalton species which tumbles slowly and therefore broadens lines, leading to considerable intensity loss in NMR spectra compared to the more rapidly tumbling dimer (50 kilodaltons) present at pH 4. Nevertheless, the combination of nearly identical NMR spectra at pH 4 with nearly identical CD spectra and similar DLS results at high salt provides considerable evidence of conserved tertiary structure in D313K relative to the WT cl-Par-4. 

The main difference observed between WT cl-Par-4 and the mutant is thus the propensity to form higher order self-associations, a propensity that is reduced for D313K at intermediate to high salt concentrations (above 250 mM NaCl or 50 mM MgSO_4_) as discussed above, but paradoxically increases at lower salt concentrations (see Figure 3). This is seen with the pH dependence as well: D313K under low salt conditions and non-optimal pH (pH 5 and pH 7) forms larger particles than does the WT (see Figure 4). It should be noted, however, that scattering techniques are highly sensitive to the largest species in a distribution of particles sizes and that uncertainty in hydrodynamics radii measurement provides some insight, but not all information, regarding particle size distribution [54]. Consequently, the DLS results by no means indicate that all particles are of the size indicated by the mean Stokes radius measurements. However, the fact remains that D313K displays an additional tendency to form at least a small number of aggregates under non-optimal salt and pH conditions. 

**Figure 8:**
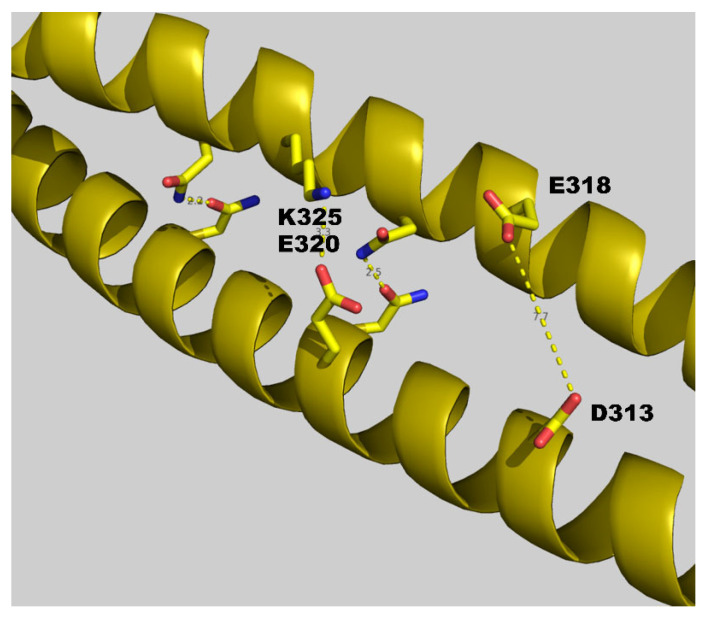
Polar interactions at the cl-Par-4 CC interface. The D313–E318 repulsion, along with the E320–K325 salt bridge and two Asn-Asn close contacts, are shown. A portion of the crystal structure of the rat Par-4 CC domain is shown, but for discussion purposes, residue numbering has been changed to coincide with the numbering of human Par-4. (PDB 5FIY, [49]).

A common theme in protein folding is that misfolding results in exposure of the hydrophobic core, which can lead to subsequent aggregation due to intermolecular hydrophobic interactions [55,56]. For a coiled coil, the hydrophobic core is not contained within one molecule, but is effectively the helix–helix dimer interface. This interface includes a leucine heptad repeat in an LZ region as well as other hydrophobic residues in both LZ and non-LZ coiled coils. A smaller number of polar residues are also typically present at or near the dimer interface. This aids folding by requiring a specific interhelical alignment in order to promote favorable charge–charge interactions and avoid unfavorable ones. This calls into question the purpose of the D313–E318 charge–charge repulsion at the Par-4 LZ interface. Other charged and polar residues at the interface would appear to contribute to stability and/or specificity. For instance, the E320–K325 interaction forms a salt bridge (see Figure 8). In addition, two asparagine residues at “a” position of the LZ should also help to properly align the helices, as they would preferentially interact with the two corresponding asparagine residues in the dimer partner, disfavoring a change of register or “sliding” of one helix relative to the other (Figure 8, unlabeled side chains).

It thus seems that the D313–E318 repulsion would be counter-productive to protein folding, and therefore its elimination and replacement with an additional K313–E318 salt bridge would stabilize the dimer and prevent aggregation. This is the case under high salt conditions (pH 7, 250 mM or higher NaCl, or 50 mM or higher MgSO_4_), but it is not the case at low salt concentration or non-optimal pH, where larger particles are formed by the mutant. It appears that unintended consequences are introduced by the D313K mutation under some conditions.

Along these lines, it should be noted that protein folding is an exquisitely balanced process. Stability of a protein to unfolding is typically around the order of 50 kJ/mol, which is closer to the strength of a hydrogen bond than to the strength of a covalent bond [57]. It is also well known that proteins from thermophiles require extra stability to withstand heat-induced unfolding and that the extra stability introduced is just sufficient to make the protein fold marginally stable in its native conditions: stability is not maximized. Even in thermophiles, stability is maintained only slightly above the unfolding threshold [58]. This is thought to be a required characteristic of proteins, allowing them to “breathe” or otherwise sample alternative conformations that may be necessary for activity. In the case of Par-4, the CC region is known to be the point of contact with coiled coil regions of important effector proteins, such as the Wilms’ tumor suppressor (WT1) [59]. If this contact is in the form of a heterodimeric CC, then some instability of the cl-Par-4 CC could assist with interactions with WT1 and other CC-containing molecules. Alternatively, the interaction may be in the form of a tetramer and thus may involve a heteromolecular analog of the cl-Par-4 tetramer that forms at high salt concentration. Again, disruption of the cl-Par-4 tetramer could then be a requirement for interaction with partners. Thus, it would be instructive to test whether the D313K mutation affects the ability of cl-Par-4 to bind WT1 and other CC-containing partners by either increasing or decreasing the stability of the cl-Par-4 tumor suppressor under conditions prevailing in the cell.

It is difficult to interpret the meaning of higher order structure formation (e.g., aggregation) in vitro as the in vivo environment contains a far lower concentration of cl-Par-4 than is required for CD or NMR spectroscopy, and the cellular environment is also filled with many other molecules and surfaces that could interact and influence self-interactions of cl-Par-4. Some of these interactions are necessary for the localization of cl-Par-4. We have mentioned that cl-Par-4 can induce apoptosis selectively in cancer cells. This selectivity has been linked to two protein–protein interactions. First, cl-Par-4 selectively enters cancer cells by binding GRP78 at the cell surface; GRP78 is expressed at higher levels in cancer cells. This interaction involves the SAC domain of cl-Par-4. In addition, the SAC domain must be phosphorylated by PKA at position T163 in order to promote nuclear entry and subsequent down-regulation of self-survival pathways. Although we have not found evidence of naturally occurring D313K mutations, this engineered mutant may be useful. It is possible that by influencing folding stability, the D313K mutation could affect the ability of cl-Par-4 to interact with GRP78 and PKA and hence could affect its ability to traverse both the cell membrane and the nuclear membrane. Any such effect upon interactions and localization would be instructive as to the mechanism of action of cl-Par-4 and could potentially lead to more selective agents against cancer cells. The results of the in vivo experiments needed to probe these possibilities will appear in due course.

## Data Availability

Not applicable.

## References

[1] Liberles D.A., Teichmann S.A., Bahar I., Bastolla U., Bloom J., Bornberg-Bauer E., Colwell L.J., de Koning A.P.J., Dokholyan N.V., Echave J. (2012). The interface of protein structure, protein biophysics, and molecular evolution. Protein Sci..

[2] Trivedi V.D. (2022). Protein structure–function exploration initiative in undergraduate biochemistry and independent research courses. Biochem. Mol. Biol. Educ..

[3] Arai M., Sugase K., Dyson H.J., Wright P.E. (2015). Conformational propensities of intrinsically disordered proteins influence the mechanism of binding and folding. Proc. Natl. Acad. Sci. USA.

[4] Manukian S., Lindberg G.E., Punch E., Mudiyanselage S.P.D., Gage M.J. (2022). pH-Dependent Compaction of the Intrinsically Disordered Poly-E Motif in Titin. Biology.

[5] Lindsay R.J., Mansbach R.A., Gnanakaran S., Shen T. (2021). Effects of pH on an IDP conformational ensemble explored by molecular dynamics simulation. Biophys. Chem..

[6] Bah A., Forman-Kay J.D. (2016). Modulation of Intrinsically Disordered Protein Function by Post-translational Modifications. J. Biol. Chem..

[7] Liu N., Guo Y., Ning S., Duan M. (2020). Phosphorylation regulates the binding of intrinsically disordered proteins via a flexible conformation selection mechanism. Commun. Chem..

[8] Wicky B.I.M., Shammas S.L., Clarke J. (2017). Affinity of IDPs to their targets is modulated by ion-specific changes in kinetics and residual structure. Proc. Natl. Acad. Sci. USA.

[9] Bondos S.E., Dunker A.K., Uversky V.N. (2022). Intrinsically disordered proteins play diverse roles in cell signaling. Cell Commun. Signal..

[10] Cheng Y., LeGall T., Oldfield C.J., Dunker A.K., Uversky V.N. (2006). Abundance of intrinsic disorder in protein associated with cardiovascular disease. Biochemistry.

[11] Du Z., Uversky V.N. (2017). A Comprehensive Survey of the Roles of Highly Disordered Proteins in Type 2 Diabetes. Int. J. Mol. Sci..

[12] Mészáros B., Hajdu-Soltész B., Zeke A., Dosztányi Z. (2021). Mutations of Intrinsically Disordered Protein Regions Can Drive Cancer but Lack Therapeutic Strategies. Biomolecules.

[13] Martinelli A.H.S., Lopes F.C., John E.B.O., Carlini C.R., Ligabue-Braun R. (2019). Modulation of Disordered Proteins with a Focus on Neurodegenerative Diseases and Other Pathologies. Int. J. Mol. Sci..

[14] Libich D.S., Schwalbe M., Kate S., Venugopal H., Claridge J.K., Edwards P.J., Dutta K., Pascal S.M. (2009). Intrinsic disorder and coiled-coil formation in prostate apoptosis response factor 4. FEBS J..

[15] Ward J.J., McGuffin L.J., Bryson K., Buxton B.F., Jones D.T. (2004). The DISOPRED server for the prediction of protein disorder. Bioinformatics.

[16] Jones D.T., Cozzetto D. (2015). DISOPRED3: Precise disordered region predictions with annotated protein-binding activity. Bioinformatics.

[17] He B., Wang K., Liu Y., Xue B., Uversky V.N., Dunker A.K. (2009). Predicting intrinsic disorder in proteins: An overview. Cell Res..

[18] Erdős G., Pajkos M., Dosztányi Z. (2021). IUPred3: Prediction of protein disorder enhanced with unambiguous experimental annotation and visualization of evolutionary conservation. Nucleic Acids Res..

[19] Ishida T., Kinoshita K. (2007). PrDOS: Prediction of disordered protein regions from amino acid sequence. Nucleic Acids Res..

[20] Burikhanov R., Zhao Y., Goswami A., Qiu S., Schwarze S.R., Rangnekar V.M. (2009). The tumor suppressor Par-4 activates an extrinsic pathway for apoptosis. Cell.

[21] Fernandez-Marcos P.J., Abu-Baker S., Joshi J., Galvez A., Castilla E.A., Cañamero M., Collado M., Saez C., Moreno-Bueno G., Palacios J. (2009). Simultaneous inactivation of Par-4 and PTEN in vivo leads to synergistic NF-κB activation and invasive prostate carcinoma. Proc. Natl. Acad. Sci. USA.

[22] Alvarez J.V., Pan T.C., Ruth J., Feng Y., Zhou A., Pant D., Grimley J.S., Wandless T.J., DeMichele A., Chodosh L.A. (2013). Par-4 Downregulation Promotes Breast Cancer Recurrence by Preventing Multinucleation following Targeted Therapy. Cancer Cell.

[23] Shelke G.V., Jagtap J.C., Kim D.-K., Shah R.D., Das G., Shivayogi M., Pujari R., Shastry P. (2018). TNF-α and IFN-γ Together Up-Regulates Par-4 Expression and Induce Apoptosis in Human Neuroblastomas. Biomedicines.

[24] Cook J., Krishnan S., Ananth S., Sells S.F., Shi Y., Walther M.M., Linehan W.M., Sukhatme V.P., Weinstein M.H., Rangnekar V.M. (1999). Decreased expression of the pro-apoptotic protein Par-4 in renal cell carcinoma. Oncogene.

[25] Guo Q., Fu W., Xie J., Luo H., Sells S.F., Geddes J.W., Bondada V., Rangnekar V.M., Mattson M.P. (1998). Par-4 is a mediator of neuronal degeneration associated with the pathogenesis of Alzheimer disease. Nat. Med..

[26] El-Guendy N., Rangnekar V.M. (2003). Apoptosis by Par-4 in cancer and neurodegenerative diseases. Exp. Cell Res..

[27] Xie J., Guo Q. (2005). PAR-4 is involved in regulation of beta-secretase cleavage of the Alzheimer amyloid precursor protein. J. Biol. Chem..

[28] El-Guendy N., Zhao Y., Gurumurthy S., Burikhanov R., Rangnekar V.M. (2003). Identification of a Unique Core Domain of Par-4 Sufficient for Selective Apoptosis Induction in Cancer Cells. Mol. Cell. Biol..

[29] Shrestha-Bhattarai T., Rangnekar V.M. (2010). Cancer-selective apoptotic effects of extracellular and intracellular Par-4. Oncogene.

[30] Gurumurthy S., Goswami A., Vasudevan K.M., Rangnekar V.M. (2005). Phosphorylation of Par-4 by protein kinase A is critical for apoptosis. Mol. Cell Biol..

[31] Chaudhry P., Singh M., Parent S., Asselin E. (2012). Prostate apoptosis response 4 (Par-4), a novel substrate of caspase-3 during apoptosis activation. Mol. Cell. Biol..

[32] Camandola S., Mattson M.P. (2000). Pro-apoptotic action of PAR-4 involves inhibition of NF-kappaB activity and suppression of BCL-2 expression. J. Neurosci. Res..

[33] Qiu G., Ahmed M., Sells S.F., Mohiuddin M., Weinstein M.H., Rangnekar V.M. (1999). Mutually exclusive expression patterns of Bcl-2 and Par-4 in human prostate tumors consistent with down-regulation of Bcl-2 by Par-4. Oncogene.

[34] Cheema S.K., Mishra S.K., Rangnekar V.M., Tari A.M., Kumar R., Lopez-Berestein G. (2003). Par-4 transcriptionally regulates Bcl-2 through a WT1-binding site on the bcl-2 promoter. J. Biol. Chem..

[35] Chen X., Sahasrabuddhe A.A., Szankasi P., Chung F., Basrur V., Rangnekar V.M., Pagano M., Lim M.S., Elenitoba-Johnson K.S. (2014). Fbxo45-mediated degradation of the tumor-suppressor Par-4 regulates cancer cell survival. Cell Death Differ..

[36] Cheratta A.R., Thayyullathil F., Pallichankandy S., Subburayan K., Alakkal A., Galadari S. (2021). Prostate apoptosis response-4 and tumor suppression: It’s not just about apoptosis anymore. Cell Death Dis..

[37] Clark A.M., Ponniah K., Warden M.S., Raitt E.M., Yawn A.C., Pascal S.M. (2018). pH-Induced Folding of the Caspase-Cleaved Par-4 Tumor Suppressor: Evidence of Structure Outside of the Coiled Coil Domain. Biomolecules.

[38] Clark A.M., Ponniah K., Warden M.S., Raitt E.M., Smith B.G., Pascal S.M. (2019). Tetramer formation by the caspase-activated fragment of the Par-4 tumor suppressor. FEBS J..

[39] Raut K.K., Ponniah K., Pascal S.M. (2021). Structural Analysis of the cl-Par-4 Tumor Suppressor as a Function of Ionic Environment. Biomolecules.

[40] van der Lee R., Buljan M., Lang B., Weatheritt R.J., Daughdrill G.W., Dunker A.K., Fuxreiter M., Gough J., Gsponer J., Jones D.T. (2014). Classification of intrinsically disordered regions and proteins. Chem. Rev..

[41] Dutta K., Engler F.A., Cotton L., Alexandrov A., Bedi G.S., Colquhoun J., Pascal S.M. (2003). Stabilization of a pH-sensitive apoptosis-linked coiled coil through single point mutations. Protein Sci..

[42] Dutta K., Alexandrov A., Huang H., Pascal S.M. (2001). pH-induced folding of an apoptotic coiled coil. Protein Sci..

[43] Edelheit O., Hanukoglu A., Hanukoglu I. (2009). Simple and efficient site-directed mutagenesis using two single-primer reactions in parallel to generate mutants for protein structure-function studies. BMC Biotechnol..

[44] Alexandrov A., Dutta K., Pascal S.M. (2001). MBP fusion protein with a viral protease cleavage site: One-step cleavage/purification of insoluble proteins. Biotechniques.

[45] Skinner S.P., Fogh R.H., Boucher W., Ragan T.J., Mureddu L.G., Vuister G.W. (2016). CcpNmr AnalysisAssign: A flexible platform for integrated NMR analysis. J. Biomol. NMR.

[46] Wuo M.G., Mahon A.B., Arora P.S. (2015). An Effective Strategy for Stabilizing Minimal Coiled Coil Mimetics. J. Am. Chem. Soc..

[47] Thayyullathil F., Pallichankandy S., Rahman A., Kizhakkayil J., Chathoth S., Patel M., Galadari S. (2013). Caspase-3 mediated release of SAC domain containing fragment from Par-4 is necessary for the sphingosine-induced apoptosis in Jurkat cells. J. Mol. Signal..

[48] Ko J., Park H., Heo L., Seok C. (2012). GalaxyWEB server for protein structure prediction and refinement. Nucleic Acids Res..

[49] Tiruttani Subhramanyam U.K., Kubicek J., Eidhoff U.B., Labahn J. (2017). Structural basis for the regulatory interactions of proapoptotic Par-4. Cell Death Differ..

[50] Felicori L., Jameson K.H., Roblin P., Fogg M.J., Garcia-Garcia T., Ventroux M., Cherrier M.V., Bazin A., Noirot P., Wilkinson A.J. (2016). Tetramerization and interdomain flexibility of the replication initiation controller YabA enables simultaneous binding to multiple partners. Nucleic Acids Res..

[51] Arakawa T., Tokunaga M., Kita Y., Niikura T., Baker R.W., Reimer J.M., Leschziner A.E. (2021). Structure Analysis of Proteins and Peptides by Difference Circular Dichroism Spectroscopy. Protein J..

[52] Greenfield N.J. (2006). Using circular dichroism spectra to estimate protein secondary structure. Nat. Protoc..

[53] Micsonai A., Wien F., Kernya L., Lee Y.-H., Goto Y., Réfrégiers M., Kardos J. (2015). Accurate secondary structure prediction and fold recognition for circular dichroism spectroscopy. Proc. Natl. Acad. Sci. USA.

[54] Stetefeld J., McKenna S.A., Patel T.R. (2016). Dynamic light scattering: A practical guide and applications in biomedical sciences. Biophys. Rev..

[55] March D., Bianco V., Franzese G. (2021). Protein Unfolding and Aggregation near a Hydrophobic Interface. Polymers.

[56] Routledge K.E., Tartaglia G.G., Platt G.W., Vendruscolo M., Radford S.E. (2009). Competition between intramolecular and intermolecular interactions in an amyloid-forming protein. J. Mol. Biol..

[57] Fersht A. (1999). Structure and Mechanism in Protein Science: A Guide to Enzyme Catalysis and Protein Folding.

[58] Razvi A., Scholtz J.M. (2006). Lessons in stability from thermophilic proteins. Protein Sci..

[59] Johnstone R.W., See R.H., Sells S.F., Wang J., Muthukkumar S., Englert C., Haber D.A., Licht J.D., Sugrue S.P., Roberts T. (1996). A novel repressor, par-4, modulates transcription and growth suppression functions of the Wilms’ tumor suppressor WT1. Mol. Cell. Biol..

