# Correction: Pandey et al. Enhancing the Conformational Stability of the cl-Par-4 Tumor Suppressor via Site-Directed Mutagenesis. *Biomolecules* 2023, *13*, 667

**DOI:** 10.3390/biom15081155

**Published:** 2025-08-12

**Authors:** Samjhana Pandey, Krishna K. Raut, Antoine Baudin, Lamya Djemri, David S. Libich, Komala Ponniah, Steven M. Pascal

**Affiliations:** 1 Biomedical Sciences Program, Old Dominion University, Norfolk, VA 23529, USA; spand003@odu.edu; 2 Department of Chemistry and Biochemistry, Old Dominion University, Norfolk, VA 23529, USA; kraut001@odu.edu (K.K.R.); ldjem001@odu.edu (L.D.); kponniah@odu.edu (K.P.); 3 Greehey Children’s Cancer Research Institute, University of Texas Health Science Center at San Antonio, San Antonio, TX 78229, USA; baudin@uthscsa.edu (A.B.); libich@uthscsa.edu (D.S.L.); 4 Department of Biochemistry and Structural Biology, University of Texas Health Science Center at San Antonio, San Antonio, TX 78229, USA

## Removal of an Author

Andrea M. Clark was included as an author in the original publication [1] and should not have been. The corrected Author Contributions statement appears here.

**Corrected Author Contributions**

Conceptualization: S.M.P.; investigation, S.P., K.K.R. and K.P.; data acquisition: S.P., K.K.R., A.B. and L.D.; writing—original draft preparation: S.P.; writing—review and editing: S.P. and S.M.P.; visualization: S.P. and K.K.R.; supervision and funding acquisition: S.M.P. and D.S.L.; resources and project administration: S.M.P. All authors have read and agreed to the published version of the manuscript.

The authors state that the scientific conclusions are unaffected. This correction was approved by the Academic Editor. The original publication has also been updated.
